# Comparison of customized spin-column and salt-precipitation finger-prick blood DNA extraction

**DOI:** 10.1042/BSR20140105

**Published:** 2014-10-22

**Authors:** Jun-Jie Poh, Samuel Ken-En Gan

**Affiliations:** *Bioinformatics Institute, Agency for Science, Technology, and Research (A*STAR), Singapore 138671, Singapore; †p53 Laboratory, Agency for Science, Technology, and Research (A*STAR), Singapore 138648, Singapore

**Keywords:** column-based purifications, gDNA extraction, nucleated blood, PCR; salt-precipitation, whole blood, dH_2_O, distilled water, gDNA, genomic DNA, RBC, red blood cell, TAE, Tris/acetate/EDTA

## Abstract

gDNA (genomic DNA extraction from blood is a fundamental process in many diagnostic, identification and research applications. Numerous extraction methods have been reported and are available commercially. However, there is insufficient understanding of the impact of chemical buffers on DNA yield from either whole or nucleated blood. Moreover, these commercial kits are often costly, constraining less well-funded laboratories to traditional and more cost-effective salt-precipitation methods. Towards this, we compared a salt-precipitation and a customized cost-effective spin-column-based method, studying the impact of different chemical constituents on the yields. This customized method resulted in a shortening of the extraction process, higher gDNA yields, and more successful PCR amplification of gDNA genes compared with the salt-precipitation method. Optimizing different chemical buffers on whole- and nucleated blood materials further revealed that certain chemicals boosted extractions from whole- but not nucleated blood. These findings may be useful to laboratories that do not have ready access to commercial kits, and improve their nucleic acid extractions from blood economically.

## Abstract 2

A customized spin-column finger-prick gDNA extraction yielded more DNA and gene amplification success than a salt-precipitation method. Further optimization showed certain buffers to augment whole, but not nucleated blood extractions.

## INTRODUCTION

Isolating high-quality nucleic acids from blood is part of many diagnostic, forensic and scientific applications ranging from: pathogen detection in livestock and humans such as HIV, HBV, HCV and bacteria [1–5]; forensics identification [6]; disease diagnosis (such as cancer) [7–10]; genetic disorders [6,11,12]; and neonatal methylation studies [13]. To meet this wide range of uses, powerful diagnostic and identification capabilities for clinical applications are in constant demand, especially if they utilize less-invasive blood extractions and are coupled with sensitive amplification methods.

The first chemical-based ‘salt-precipitation’ method for DNA extraction from blood was reported by Miller [14]. This involved the use of saturated NaCl–ethanol solutions on nucleated cells. These are obtained by subjecting whole blood to RBC (red blood cell) lysis buffers, resulting in erythrocyte depletion. While the salt-precipitation methods for gDNA extraction are efficient, they are laborious and time-consuming, leading to the adoption of silica columns, which became popular for general nucleic acid extraction due to their convenience and ease of use. Spin-column kits gradually became the new gold standard for commercial blood gDNA extraction. Amongst the commonly available brands, QIAGEN, in particular, has shown to have columns with superior binding capability [13].

Nonetheless, the salt-precipitation method is economically more viable, allows more control over the parameters involved [15], and reportedly giving better yields [16] than commercial kits (e.g. QIAGEN). With these different options, the ideal conditions for gDNA extractions in these technologies require attention.

In this report, we compared a salt-precipitation method with a customized spin-column method (adapted from a bacterial plasmid miniprep kit), and studied the chemical buffer factors that influence the gDNA yield from finger-pricked whole and processed nucleated blood cells. With a view towards economic and efficient gDNA extraction from blood, and to determine the quality of these extractions, PCR amplification of antibody variable (v)-region genes from the extracted nucleic acids were assessed as a proof-of-concept for DNA-based diagnosis.

## MATERIALS & METHODS

### Salt-precipitation protocol

The selected salt-precipitation protocol on whole blood was performed according to the ‘Comparative and Molecular Pharmacogenomic Lab’ in Tufts University, USA [15]. Briefly, finger-prick whole blood was incubated with cold sterile dH_2_O (distilled water) for 2–3 min, followed by centrifugation at 3.5 k rpm for 15 min. The pellet was resuspended in RBC lysis buffer A and water, with quick vortex to lyse the erythrocytes. The suspension was then spun, and the lysis step repeated. Lysis buffer B (for nucleated cells) and 10% SDS (w/v) were added to the pellet and mixed. The mixture was then incubated for 2 h at 55°C, cooled on ice, thoroughly mixed with 5.3 M NaCl by vortexing, and pelleted at 4.5 k rpm for 20 min. The recovered supernatant was gently mixed with an equal volume of −20°C isopropanol. Precipitated DNA pellets were removed and transferred to microfuge tubes where they were washed with 70% (v/v) ethanol, dried, and resuspended in dH_2_O or TE buffer.

### Customized column-based DNA extraction method

The column-based DNA extraction was adapted using the first three steps of the salt-precipitation method [15] followed by the use of a plasmid miniprep kit (Quintech Life Sciences Pte Ltd). Briefly, whole blood was processed in accordance to the salt precipitation method till the third step when cells were treated with lysis buffer B [15]. The solutions were spun at 13 k rpm for 10 min prior to the transfer of supernatant to equilibrated Quintech miniprep kit spin-columns [17]. Equilibration was performed by adding equilibration buffer BK to the column to stand for 2 min before spinning it out at max speed. The blood lysate was then added to the column, allowed to stand for 2 min before adding the pre-wash buffer (W1-to remove endonucleases). After spinning out W1, wash buffer W2 was added to the column followed by a dry spin to dry the column. The DNA were eluted using EB buffer. All subsequent steps were performed according to the manufacturer's recommendations.

The comparison of the salt-precipitation and the adapted column-based methods were performed using finger-prick blood from volunteers. A minimum of 0.5 ml of blood were taken using lancets (VWR International) and separated into equal volumes for triplicate column-based and salt-precipitation protocol comparisons.

### Optimization of DNA extraction for nucleated- and whole blood

To study the effect of chaotrophic buffers on nucleated cell DNA extraction, the supernatant from step 3 (containing the lysed nucleated cells) of the salt-precipitation protocol [15] were premixed with pre-wash/protein-binding buffers W1/QG2 [17] in 4:1 (v/v) ratio prior to transfer onto an equilibrated spin-column. These were compared with the above customized column-based method where the lysates were directly applied onto the equilibrated column. Subsequent steps of DNA extractions were performed according to the column-based protocol.

To study the effect of chaotropic buffers on whole-blood DNA extraction, 20 μl of salt-precipitation buffer B and 100 μl of PBS were added to 100 μl of whole blood (step is adapted from QIAGEN's DNeasy® Blood & Tissue kit protocol). The solutions were then subjected to steps 3 and 4 of the salt-precipitation method [15] before premixing with relevant chaotropic buffer at the application step to the spin-column as performed for the nucleated blood DNA extraction. For consistency, 100 μl of blood was used as the starting material for all comparisons between nucleated and whole-blood. All DNA readings were measured in triplicates using the IMPLEN Nanophotometer P330.

### Gene amplification from extracted DNA

Suitability of the extracted nucleic acid for further analysis were assessed using PCR amplifications of antibody v-regions using primers targeting the signal peptide and constant regions, previously described to yield ~350–400 bp PCR products [18]. The identity of the products were sequence analysed using DNAApp v 1.2 [19] and IgBlast [20].

## RESULTS & DISCUSSION

Comparing the gDNA yields from finger-prick blood using a customized spin-column and a salt-precipitation method, we found that the former increased the ease, speed of the process, and also the success of subsequent PCR amplification experiments. Further optimization of our adapted method revealed certain chaotropic buffers to affect the yields from whole- or erythrocyte depleted nucleated blood cells.

**Figure 1:**
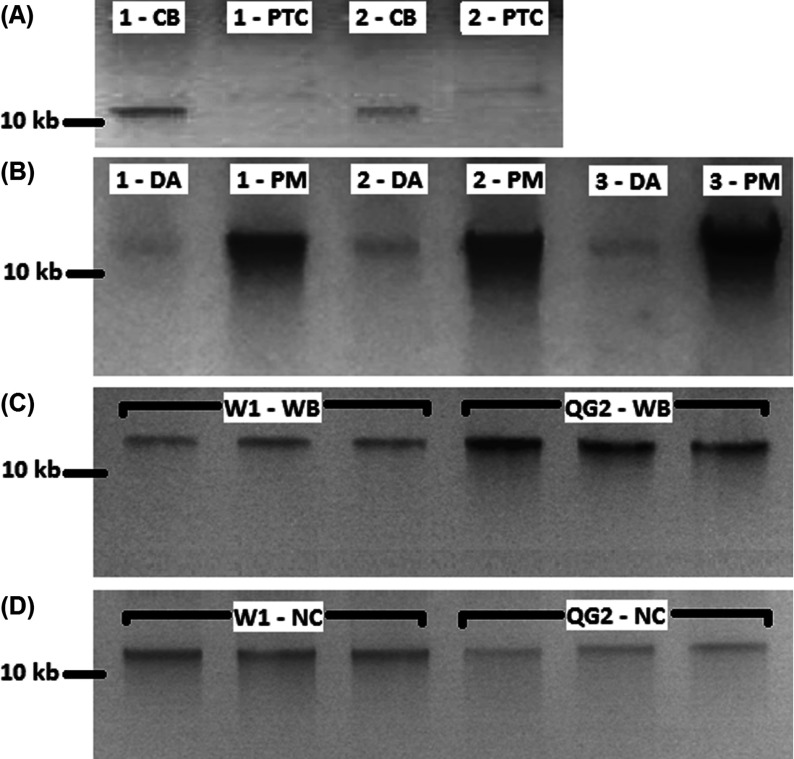
Gel comparison of various blood genomic DNA extraction methods (**A**) Comparison of column-based and salt-precipitation blood extraction methods. Numbers denote the volunteer for the finger-prick blood samples. CB, column-based blood extraction; PTC, salt-precipitation blood extraction protocol. (**B**) Comparison of premixed with W1, and direct application of cell lysate column-based blood extraction methods. Numbers denote the volunteer for the finger-prick blood samples. DA, direct application of cell lysate on column; PM, buffer W1 premixed with cell lysate prior to application on column. (**C**) Comparison of buffer W1 and QG2 on whole blood column-based extraction method. W1-WB, whole blood column-based extraction method premixed with buffer W1; QG2-WB, whole blood column-based extraction method premixed with buffer QG2, Extractions were carried out in triplicates. All DNA concentrations determined using IMPLEN Nanophotometer P330 were significantly different between groups in independent *T* test (*t* (16)=6.75; *P*=0.000), (**D**) Comparison of buffer W1 and QG2 on nucleated blood column-based extraction methods. W1-NC, nucleated blood column-based extraction method premixed with buffer W1, QG2-NC, nucleated blood column-based extraction method premixed with buffer QG2. Extractions were carried out in triplicates. All DNA concentrations determined using IMPLEN Nanophotometer P330 were significantly different between groups in independent *T* test (*t* (16)=6.32; *P*=0.000), 20 μl of DNA extracted were mixed with 6× loading dye and loaded on a 1% (w/v) TAE (Tris/acetate/EDTA) agarose gel. Samples were electrophoresed and visualized in RunVIEW by Cleaver Scientific. *Spectrophotometer readings were not shown as the majority were not detectable.

Conveniently, the column-based method utilized the following steps: lysis of cells; binding of DNA to the silica in the column; and elution. In contrast, the salt-precipitation protocol method involves lysis of the cells, and multiple washings steps followed by salt-precipitation of the DNA. The salt-precipitation method, although cheaper (<USD $0.5 per sample), is laborious and time consuming, resulting from the several incubation steps and long high-speed centrifugations. To overcome this, we adapted the commonly available and cost-effective plasmid DNA miniprep kit (~USD $1 per reaction) for gDNA extraction. This greatly enhanced the amount of DNA extracted (Figure 1A and Table 1A); in contrast, other reports have suggested that salt-precipitation protocol resulted in higher DNA yields [16]. Although our spectrophotometer could only detect salt-precipitated DNA from two donors (total three donors, see Table 1A), gel analysis (Figure 1A) confirmed that our column-based extractions had higher yields.

**Table 1:** Comparisons of the various methods and their optimizations for whole and nucleated blood avg, average; n.d., not detected.

(a) Comparison of spin-column method and salt-precipitated DNA extractions from blood					
Donor	Number of repeats	Column method avg [DNA] (ng/μl)	Column method avg 260/280 ratio	Salt-precipitated avg [DNA] (ng/μl)	Salt-precipitated avg 260/280 ratio
1	3	12.67±1.89	1.77±0.02	11.67±1.61	2.01±0.09
2	3	n.d.	n.d.	24.33±0.76	1.93±0.05
3	3	n.d.	n.d.	16.33±0.29	1.85±0.03
**(b) Comparison of premix with chaotropic buffer and directly applied column extractions**					
**Donor**	**Number of repeats**	**Premixed with W1 avg [DNA] (ng/μl)**	**Premixed with W1 avg 260/280 ratio**	**Direct application of W1 avg [DNA] (ng/μl)**	**Direct application of W1 avg 260/280 ratio**
1	3	23.17±2.75	1.78±0.03	n.d.	n.d.
2	3	48.33±1.15	1.82±0.02	n.d.	n.d.
3	3	60.17±1.16	1.80±0.00	n.d.	n.d.
**(c) Comparison of chaotropic buffers on whole blood column-based DNA extractions**					
**Donor**	**Number of repeats**	**Premixed with W1 avg [DNA] (ng/μl)**	**Premixed with W1 avg 260/280 ratio**	**Premixed with QG2 avg [DNA] (ng/μl)**	**Premixed with QG2 avg 260/280 ratio**
1	3	2.30±0.10	0.82±0.19	8.40±2.55	2.91±0.50
2	3	1.9±0.96	0.94±0.08	7.80±1.83	5.4±3.83
3	3	1.67±0.49	1.16±0.29	6.5±3.27	5.01±5.36
**(d) Comparison of chaotropic buffers on nucleated blood column-based DNA extractions**					
**Donor**	**Number of repeats**	**Premixed with W1 avg [DNA] (ng/μl)**	**Premixed with W1 avg 260/280 ratio**	**Premixed with QG2 avg [DNA] (ng/μl)**	**Premixed with QG2 avg 260/280 ratio**
1	3	7.73±1.17	1.50±0.10	4.93±0.40	1.46±0.15
2	3	7.23±0.25	1.77±0.14	5.03±0.70	1.62±0.10
3	3	6.8±1.13	1.65±0.23	5.30±0.45	1.58±0.04

To further optimize our column-based yields, we premixed the cell-lysate with a chaotropic (pre-wash) buffer W1 [17] prior to the application on the spin-columns. This boosted the DNA yields further. Extractions that utilized direct application of lysates were undetectable by spectrophotometry (Table 1B) and showed faint bands with gel electrophoresis (Figure 1B). On the other hand, when the cell lysate was premixed with chaotropic buffers, higher quality DNA were obtained (Table 1B). These readings were supported by the observation of intense bands (Figure 1B). Chaotropic agents (guanidinium thiocyanate) in these buffers would have played a role to remove DNA-binding proteins [17], allowing for better absorption to the spin-columns [21]. Given that the chaotropic buffer was chemically similar to many agarose gel dissolving and PCR purification buffers, they would facilitate the removal of other interfering chemicals present in whole blood.

**Figure 2:**
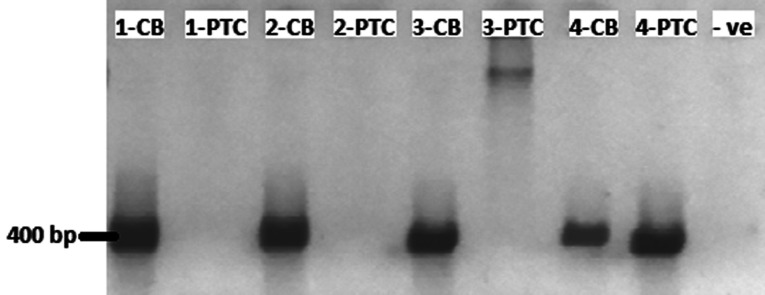
PCR of antibody v-regions of the heavy-chain on both column-based and salt-precipitation blood extraction protocol Numbers denote the volunteer for the finger-prick blood samples. CB, column-based blood extraction; PTC, salt-precipitation blood extraction; −VE, negative control, 10 μl of PCR samples were mixed with 6× loading dye and loaded on a 1% (w/v) TAE agarose gel. Samples were electrophoresed and visualized in RunVIEW by Cleaver Scientific.

To determine the suitability of the extracted nucleic acid for further downstream analyses, PCR of antibody v-regions were performed on both salt-precipitated and column-purified DNA. Figure 2 shows bands of ~400 bp in all four column-based extraction, whereas the band was only visible in one salt-precipitated gDNA (lane 8). Subsequent sequencing and verification in IgBlast [20] confirmed these bands to be antibody v-regions (data not shown). This shows that our customized column-based method boosted the success rate of PCR amplification when compared with traditional salt-precipitation methods with the same starting amount of blood.

We next optimized the buffer conditions for whole blood and nucleated blood. In a previous study, we found pre-wash buffer QG2 (high concentration chaotropic buffer) to be better than pre-wash buffer W1 (low-concentration chaotropic buffer) for gel and PCR extraction [17]. Interestingly, the use of W1 was found to give better gDNA yields on nucleated blood, whereas buffer QG2 (1 M higher chaotropic agent) was more suitable for whole blood (Figures 1C and 1D, see also Tables 1C and 1D). The higher concentration of guanidinium compounds in buffer QG2 was a likely major contributing factor. Since EDTA and Tris were present in the buffer, nuclease cofactors would be chelated while maintaining the essential pH buffering. It is possible that other additional chemicals in buffer QG2 may have perturbed the binding of gDNA to the membrane. However, experiments to further explore the effect of various components are limited by the high variability in blood samples and the low volume of blood that can be drawn from finger-pricks (also a main factor in our low [DNA] measurements and poor 260/280 ratios in Tables 1C and 1D).

For nucleated blood, the poorer performance of buffer QG2 may have resulted from the presence of excess chelating agents that had possibly removed the necessary divalent cations needed for gDNA silica binding [22]. In contrast, the isopropanol containing buffer W1, consisting of suitable amounts of chelating agents and chaotropic agents, may explain the higher yields obtained from nucleated blood.

Through the adaptation of a plasmid miniprep kit and a salt-precipitation method, we have customized a column-based protocol that is significantly more cost-effective (~USD$1 per reaction) compared with the QIAGEN Blood and Tissue kit (~USD$7 per reaction). Our customizations not only shortened the process of salt-precipitation methods, but also increased the chances of PCR gene amplification. In our optimization, we found blood DNA extractions to contrast gel and PCR extraction kits on the requirement of chelating agents and chaotropic agents. This is especially observed for erythrocyte-depleted nucleated blood.
